# A Simple and Ultrasensitive Colorimetric Biosensor for Anatoxin-a Based on Aptamer and Gold Nanoparticles

**DOI:** 10.3390/mi12121526

**Published:** 2021-12-08

**Authors:** Duy-Khiem Nguyen, Chang-Hyun Jang

**Affiliations:** Department of Chemistry, Gachon University, Seongnam-daero 1342, Sujeong-gu, Seongnam-si 13120, Korea; khiem80@gachon.ac.kr

**Keywords:** anatoxin-a, colorimetric aptasensors, aptamer, UV/Vis absorbance, gold nanoparticles

## Abstract

Here, we designed a simple, rapid, and ultrasensitive colorimetric aptasensor for detecting anatoxin-a (ATX-a). The sensor employs a DNA aptamer as the sensing element and gold nanoparticles (AuNPs) as probes. Adsorption of the aptamer onto the AuNP surface can protect AuNPs from aggregation in NaCl solution, thus maintaining their dispersion state. In the presence of ATX-a, the specific binding of the aptamer with ATX-a results in a conformational change in the aptamer, which facilitates AuNP aggregation and, consequently, a color change of AuNPs from red to blue in NaCl solution. This color variation is directly associated with ATX-a concentration and can be easily measured using a UV/Vis spectrophotometer. The absorbance variation is linearly proportional to ATX-a concentration across the concentration range of 10 pM to 200 nM, with a detection limit of 4.45 pM and high selectivity against other interferents. This strategy was successfully applied to the detection of ATX-a in lake water samples. Thus, the present aptasensor is a promising alternative method for the rapid detection of ATX-a in the environment.

## 1. Introduction

Anatoxin-a (ATX-a) is a natural organophosphate neurotoxin, produced by various cyanobacteria including *Aphanizomenon* *gracile*, *Oscillatoria acuminata*, and *Anabaena flos-aquae*, which occur naturally in freshwater [1,2]. This toxin can irreversibly bind to nicotinic acetylcholine receptors, leading to disruption of oxygen supply to the brain, causing respiratory paralysis, acute asphyxia, and ultimately death [2,3]. Exposure to ATX-a primarily occurs through the ingestion of contaminated water [1,3]. In recent years, an increase in animal intoxication due to ATX-a has been observed [2]. Thus, the development of a sensitive and accurate method for ATX-a detection is urgently needed to monitor and control the quality of both environmental water and drinking water.

To date, only a limited range of analytical techniques have been developed for ATX-a detection, such as electrochemistry [1], liquid chromatography–mass spectrometry (LC-MS/MS) [2], high-performance liquid chromatography (HPLC) [4], gas chromatography–mass spectrometry (GC–MS) [5], fluorescence [6], and electrochemiluminescence [7]. However, these techniques have several limitations including bulk and expensive instrumentation, complex sample preparation, time-consuming procedures, and the need for highly skilled personnel for operation [6,7]. Therefore, a much simpler, cost-effective, and precise alternative needs to be urgently developed for ATX-a detection.

In recent years, aptamer-based colorimetric sensing methods have emerged as alternatives to detect various target molecules [8,9,10,11,12,13], because of their simplicity, low cost, high sensitivity, and specific detection without the requirement of complex instrumentation [14]. In this assay, aptamers (single-stranded DNA or RNA oligonucleotides) are used as recognition elements owing to their ability to selectively bind their target molecules, which helps improve the sensor specificity [15,16,17,18,19,20]. Furthermore, aptamers have the distinctive advantages of high stability, high selectivity, and cost-effectiveness [21]. An aptamer for ATX-a was previously selected, characterized, and used as a recognition probe to develop a simple impedimetric aptasensor for ATX-a sensing [1].

In colorimetric sensors, gold nanoparticles (AuNPs) are commonly used as colorimetric reporters because of their high extinction coefficients and size-dependent optical properties [8,22]. The color of the dispersed AuNP solution is wine-red. However, aggregation of AuNPs increases the size of the particles, resulting in remarkable color changes of the solution from wine-red to purple or blue, which can be observed by the naked eye [22,23,24]. In addition, AuNPs have been used in combination with other nanomaterials (e.g., reduced graphene oxide) or conjugated with aptamers to improve the performance of the sensors or to enhance the detection sensitivity [25,26,27]. Moreover, AuNPs can adsorb unfolded aptamers through electrostatic and noncovalent interactions, which protects AuNPs from salt-induced aggregation by enhancing the electrostatic repulsion between nanoparticles [8,22,28]. However, when aptamers bind to their target molecules, their conformation changes. The exposure of nucleobases on aptamers is reduced, and a negatively charged phosphate backbone is presented. This leads to poor adsorption of the aptamers onto the AuNP surface; therefore, the target-bound aptamers cannot prevent the aggregation of AuNPs in solutions with high salt concentration, resulting in color changes in the nanoparticle solution [24,29]. Recently, colorimetric biosensors were integrated with a smartphone for the development of personalized point-of-care (POC) devices that can be used for self-monitoring of physical health parameters [30]. For example, Qing et al. developed a colorimetric assay using a smartphone RGB camera for self-monitoring of diabetes [31].

**Figure 1:**
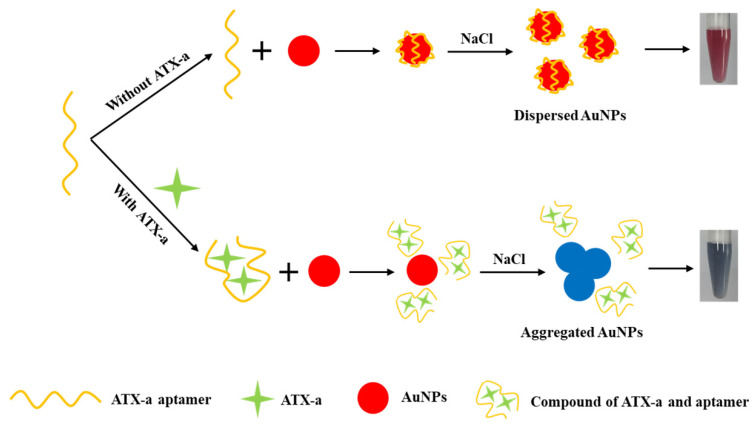
Schematic illustration of the colorimetric aptasensor for anatoxin-a (ATX-a) detection based on gold nanoparticle aggregation under high NaCl concentration.

In this study, we developed a simple, label-free colorimetric biosensing system for the sensitive detection of ATX-a using DNA aptamers as the target recognition elements and gold nanoparticles as colorimetric reporters. In the absence of ATX-a, the random coil aptamer adsorbs onto the surface of citrate-capped AuNPs and protects them from aggregation in NaCl solution; thus, the AuNPs maintain their dispersion state and the solution remains red in color (Figure 1). In the presence of ATX-a, the specific binding of aptamers with ATX-a alters the conformation of the aptamer, which reduces the adsorption of the aptamer on the AuNP surface, leading to AuNP aggregation and, consequently, a color change of the solution from red to blue under high NaCl concentration. By measuring the color and absorbance change of the AuNP solution, the concentration of ATX-a can be easily quantified. Our results indicate that the proposed colorimetric aptasensor platform can detect ATX-a in aqueous solutions at the picomolar level (4.45 pM) with high selectivity against other interferents. Furthermore, the aptasensor is simple and easy to operate, and it does not require expensive and sophisticated instruments.

## 2. Materials and Methods

### 2.1. Materials and Apparatus

The anatoxin-a-specific aptamer with the sequence 5′–TGG CGA CAA GAA GAC GTA CAA ACA CGC ACC AGG CCG GAG TGG AGT ATT CTG AGG TCG G–3′ was purchased from Mbiotech (Hanam, South Korea). Anatoxin-a fumarate salt (ATX-a), aflatoxin (AFT), cylindrospermopsin solution (CYN), microcystin-LR solution (MC-LR), HAuCl_4_·3H_2_O, sodium citrate (Na_3_C_6_H_5_O_7_·2H_2_O), calcium chloride (CaCl_2_), magnesium chloride (MgCl_2_), potassium chloride (KCl), and sodium chloride (NaCl) were obtained from Sigma-Aldrich (St. Louis, MO, USA). HCl (36%) and HNO_3_ (70%) were obtained from Daejung Chemicals & Metals Co., Ltd. (Daejung, South Korea). Citrate-coated AuNPs (15 nm mean diameter) with a concentration of approximately 4.7 nM were synthesized using the citrate reduction method as reported previously [32]. Deionized (DI) water from a Milli-Q water purification system (Millipore, CA, USA) was used to prepare the aqueous solutions.

The shape, size, and aggregation of AuNPs were examined by transmission electron spectroscopy (TEM, Tecnai G2 F30 S-TWIN) at 300 kV. UV/Vis absorption spectra were recorded using a UV/Vis spectrophotometer (Cary 50, Varian).

### 2.2. Optimization of Detection Conditions

To optimize the NaCl concentration, NaCl solutions with different concentrations were mixed with the AuNP solution and incubated for 5 min at 25 °C. The total volume of the mixture was 1.5 mL. The final concentrations of NaCl were 0, 10, 20, 30, 40, 50, and 60 mM. The absorbance value at 524 nm of each sample was then measured using a UV/Vis spectrophotometer.

To optimize the aptamer concentration, different concentrations of aptamer (0, 50, 100, 125, 150, 175, and 200 nM) were mixed with the AuNP solution and incubated for 2 h at 25 °C. NaCl solution (1.5 M) was then added, and the mixtures were incubated for 5 min at 25 °C. The total volume of the mixture was 1.5 mL, and the final concentration of NaCl was 40 mM.

### 2.3. Colorimetric Detection of ATX-a

In a typical experiment, 60 µL of 3 µM aptamer solution and 60 µL of ATX-a solution at different concentrations (final ATX-a concentrations were 0, 0.01, 0.1, 1, 10, 100, and 200 nM) were mixed in a 1.5 µL centrifuge tube and incubated at 37 °C for 2 h. Next, 1340 µL of AuNP solution was added to the assay solution, mixed well, and incubated for 2 h at room temperature (~25 °C). Finally, 40 µL of 1.5 M NaCl solution was added to the centrifuge tube at room temperature (~25 °C). After 5 min, 1.5 mL of the resulting solution was transferred to a quartz cuvette, and the absorbance of the solutions was recorded. The absorbance values of each sample at 524 nm (A_524_) were determined, and the difference between the absorbance of samples with ATX-a and the absorbance of the blank sample at 524 nm (ΔA_524_) was calculated.

To test the specificity of the present aptasensor, some common interferents such as aflatoxin (AFT), cylindrospermopsin (CYN), microcystin-LR (MC-LR), CaCl_2_, MgCl_2_, and KCl were also tested.

## 3. Results and Discussion

### 3.1. Feasibility of the Colorimetric Assay for ATX-a Detection

**Figure 2:**
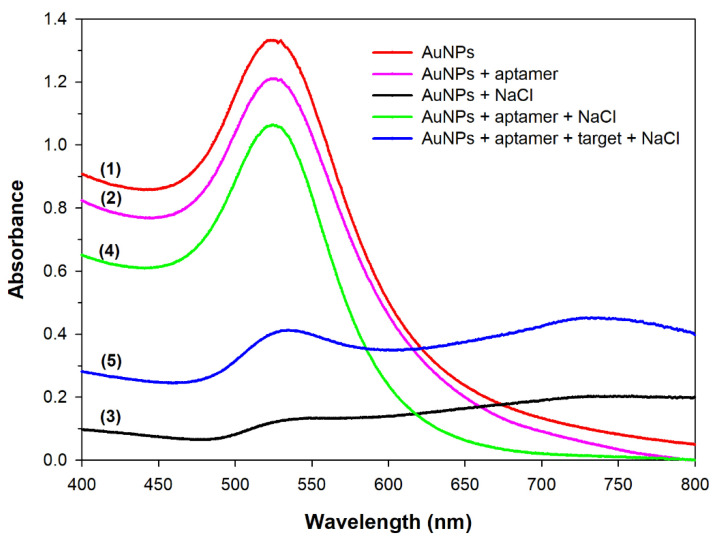
UV/visible absorption spectra of gold nanoparticle (AuNP) solutions after mixing and incubating with different substances: (1) pure AuNPs, (2) AuNPs + anatoxin-a (ATX-a) aptamer, (3) AuNPs + NaCl, (4) AuNPs + ATX-a aptamer + NaCl, and (5) AuNPs + ATX-a aptamer + ATX-a + NaCl.

The AuNPs synthesized by citrate reduction of HAuCl_4_ are wine-red in color and dispersed well in an aqueous solution owing to the electrostatic repulsion produced by the negative charges of citrate anions coated on the AuNP surface [14,28]. To prove the feasibility of this assay for ATX-a detection using AuNPs as colorimetric probes, the UV/visible absorption spectra of AuNP solutions before and after treatment with different substances were recorded. The absorption spectrum of pure AuNPs had a strong absorbance peak at 524 nm (curve 1 in Figure 2), indicating that the AuNPs were well dispersed in the solution. However, after incubation with 40 mM NaCl, the intensity of the absorbance peak at 524 nm dramatically decreased, implying that the AuNPs aggregated (curve 3 in Figure 2). This is due to the negative charges of citrates coated on the AuNP surface being neutralized by Na^+^ ions, leading to a reduction in electrostatic repulsion and subsequent AuNP aggregation [9,24]. DNA aptamers have been reported to be adsorbed onto the AuNP surface, thus enhancing the electrostatic repulsion between nanoparticles, and protecting AuNPs from NaCl-induced aggregation [22,33]. Therefore, upon aptamer addition to the AuNP solution, the intensity of the absorbance peak at 524 nm decreased only slightly compared to that of pure AuNPs, indicating that the AuNPs were still dispersed (curve 4 in Figure 2). However, upon the addition of both aptamers and ATX-a, the absorbance at 524 nm decreased drastically, indicating the aggregation of AuNPs (curve 5 in Figure 2). The ATX-a aptamer undergoes a conformational change induced by the specific binding of aptamers and ATX-a. This change reduces the aptamer adsorption on the AuNP surface, thus diminishing the protective effect of the aptamer and leading to AuNP aggregation [1,9].

**Figure 3:**
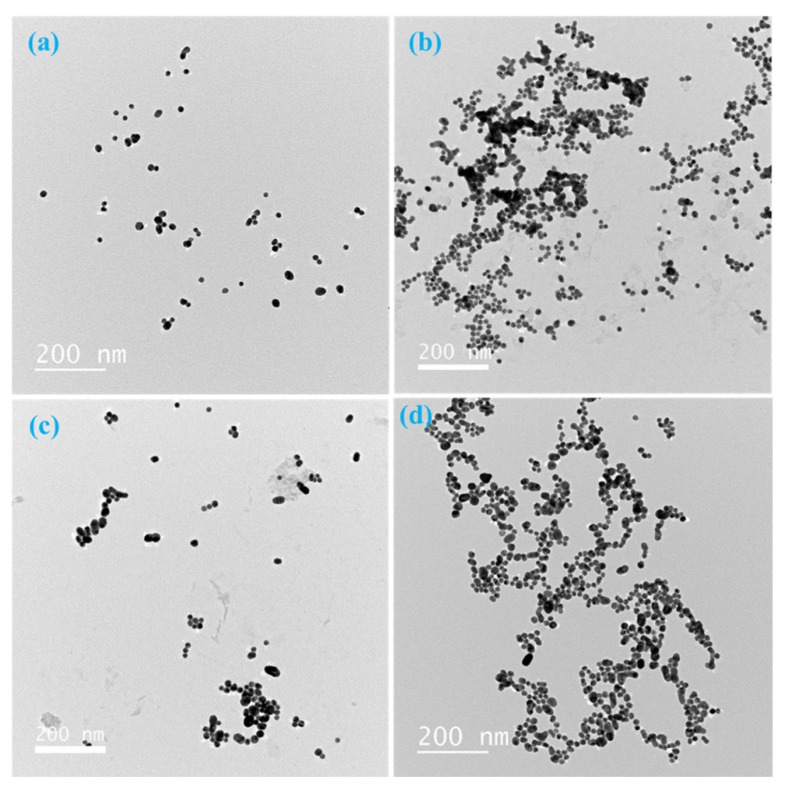
Transmission electron microscopy (TEM) images of gold nanoparticles (AuNPs) in different systems: (**a**) pure AuNPs, (**b**) AuNPs + 40 mM NaCl, (**c**) AuNPs + 125 nM anatoxin-a (ATX-a) aptamer + 40 mM NaCl, and (**d**) AuNPs + 125 nM ATX-a aptamer + 100 nM ATX-a + 40 mM NaCl.

The aggregation states of AuNPs under different conditions were further confirmed by transmission electron spectroscopy (TEM). Figure 3a shows that the free AuNPs were well dispersed in the solution and had a spherical morphology with a mean diameter of 15 nm. Addition of 40 mM NaCl caused complete aggregation of the AuNPs (Figure 3b). In the presence of the aptamer, the AuNPs aggregated slightly after 5 min of incubation with 40 mM NaCl (Figure 3c). However, in the presence of both the aptamer and ATX-a, obvious aggregation of AuNPs was observed in the presence of 40 mM NaCl (Figure 3d). These results are consistent with the results obtained from UV/Vis absorption spectra.

### 3.2. Optimization of the Detection Conditions

**Figure 4:**
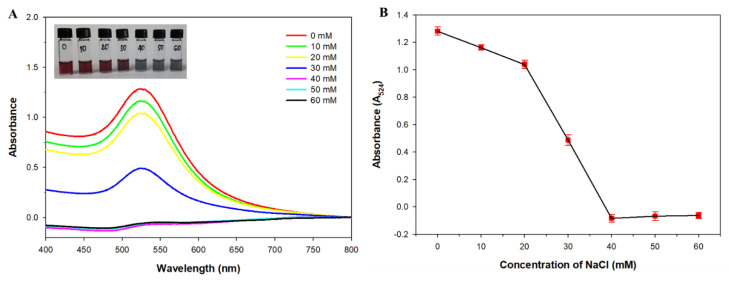
The UV/Vis absorption spectra (**A**) and absorbance intensity changes at 524 nm (**B**) of the gold nanoparticle (AuNP) solutions in the presence of various concentrations of NaCl. Inset: visible color changes of the AuNP solutions.

As NaCl causes AuNP aggregation and directly affects the performance of the colorimetric assay, the concentration of NaCl was first optimized. Figure 4A shows that the absorbance of the AuNP solution at 524 nm gradually decreased and the color of the solutions changed from wine-red to blue (inset of Figure 4A) as the NaCl concentration increased. The lowest absorbance intensity was achieved with 40 mM NaCl, suggesting that the AuNPs were completely aggregated (Figure 4B). Thus, 40 mM NaCl was used for the subsequent experiments.

**Figure 5:**
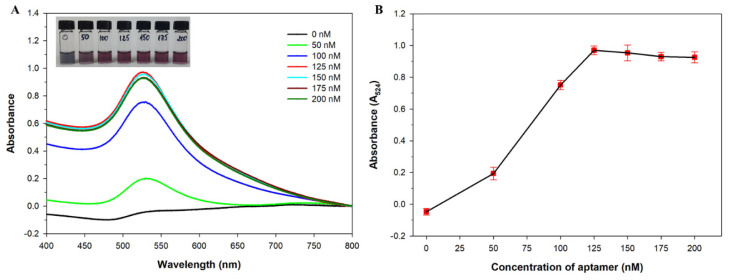
The UV/Vis absorption spectra (**A**) and absorbance intensity changes at 524 nm (**B**) of the gold nanoparticle (AuNP) solutions containing different concentrations of anatoxin-a aptamer after the addition of 40 mM NaCl. Inset: visible color changes of AuNP solutions.

Aptamer concentration is also an important factor that affects the sensitivity and performance of colorimetric assays. To obtain the optimal aptamer concentration, different concentrations of the aptamer in the range of 0–200 nM were mixed with the AuNP solution and incubated for 2 h at 25 °C, followed by the addition of 40 mM NaCl solution. The result showed that the absorbance intensity at 524 nm reached the maximum value at an aptamer concentration of 125 nM, suggesting the highest protective effect of the aptamer against NaCl-induced aggregation (Figure 5). Therefore, the optimal aptamer concentration was 125 nM.

Incubation time is also an important factor to be considered because it can affect the performance of the biosensor. As illustrated in Figure S1, the optimal incubation time of aptamer with AuNPs was 2 h, and the optimal binding time of aptamer with ATX-a was 2 h.

The adsorption of the DNA aptamer onto the AuNP surface may be affected by the pH value of the solution. A previous study demonstrated that the highest sensitivity of the sensor was observed at the pH value of 7.0 [11]. Therefore, in our proposed colorimetric assay, the detection of anatoxin-a was performed at pH 7.0.

### 3.3. Sensitivity, Specificity, Repeatability, and Stability of the Colorimetric Assay for ATX-a Detection

**Figure 6:**
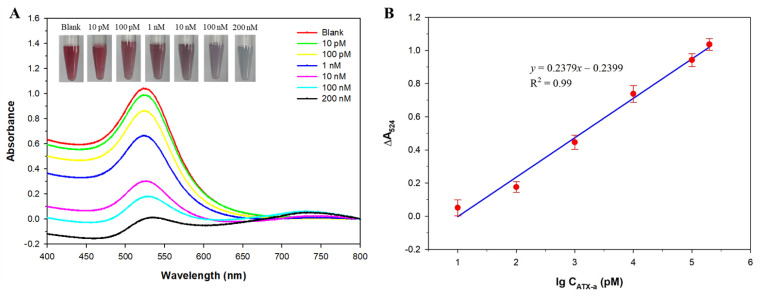
(**A**) The UV/Vis absorption spectra of the gold nanoparticle (AuNP) solutions in the presence of mixtures of 125 nM aptamer and various concentrations of anatoxin-a (ATX-a) after incubation with 40 mM NaCl for 5 min (inset: visible color changes of AuNP solutions). (**B**) Derived calibration curve showing the variation of relative absorbance at 524 nm (ΔA_524_) versus the logarithmic concentration of ATX-a.

The proposed colorimetric aptasensor was then applied for ATX-a detection under the optimized experimental conditions. A series of varying concentrations of ATX-a was added, and the UV/Vis absorption spectra of the solutions were recorded. The relationship between the absorbance variation (ΔA_524_) and ATX-a concentration was then investigated. As presented in Figure 6A, the color of the AuNP solutions gradually changed from wine-red to purple and blue (inset of Figure 6A), and the absorbance of AuNPs at 524 nm gradually decreased with increasing ATX-a concentration. The absorbance variation was linearly proportional to the logarithmic concentration of ATX-a across the concentration range of 10 pM to 200 nM (Figure 6B). The linear regression equation was ΔA_524_ = 0.2379 log C_ATX-a_ − 0.2399 (*R*^2^ = 0.99). The detection limit (LOD) of ATX-a was calculated to be 4.45 pM, which was determined using the equation 3α/m, where α is the standard deviation of blank and m is the slope of the calibration curve [26,34]. Compared to other existing techniques for ATX-a detection such as HPLC, GC–MS, electrochemistry, fluorescence, and electrochemiluminescence, the proposed colorimetric assay exhibits a wider linear range and a lower LOD for ATX-a, as summarized in Table 1.

**Figure 7:**
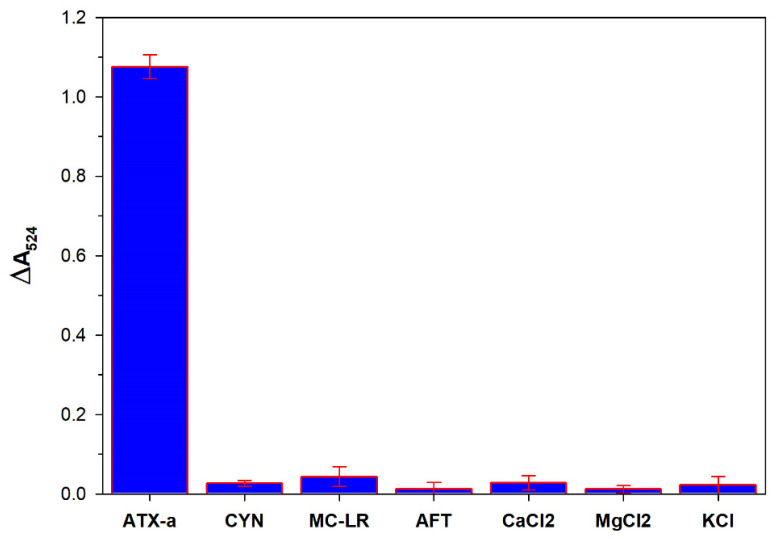
Specificity of the proposed colorimetric aptasensor for anatoxin-a (ATX-a). The ATX-a concentration was 200 nM. The concentration of other interferents was 1 µM.

The specificity of the proposed biosensor was investigated. To test specificity, ATX-a (200 nM) and other common interferents (all at 1 µM) such as aflatoxin (AFT), cylindrospermopsin (CYN), microcystin-LR (MC-LR), CaCl_2_, MgCl_2_, and KCl were tested under the same optimized experimental conditions. As illustrated in Figure 7, the change in absorbance at 524 nm for the target ATX-a was significant, whereas it was negligible for the other interferents. The results indicated that the proposed colorimetric aptasensor has excellent specificity toward ATX-a, which can be attributed to the highly specific binding of ATX-a aptamers with ATX-a.

Five replicate measurements of ATX-a (200 nM) were carried out under the optimal conditions to check the repeatability of the developed sensor. The relative standard deviation was 3.6%, suggesting excellent repeatability.

To assess the stability of the developed sensor, the synthesized AuNPs were stored at room temperature (~25 °C) and used to determine ATX-a once a day. After 10 days, the absorbance value of 200 nM ATX-a remained stable, and the relative standard deviation was <5%, indicating excellent stability of the developed sensor for ATX-a detection.

### 3.4. Analysis of Real Samples

The performance of the developed colorimetric aptasensor for the detection of ATX-a in an actual water environment was then demonstrated. The natural water sample was obtained from a local lake (Shin Dae Lake, Suwon City, Republic of Korea), and different ATX-a concentrations were used to spike the samples, followed by analysis using the developed assay. The results, as summarized in Table 2, show that the recoveries were in the range of 89.72% to 112.43%, and that the relative standard deviation (RSD) was between 4.12% and 10.91%, indicating the acceptable accuracy of the developed aptasensor for ATX-a detection. These results demonstrate that the present biosensor has potential for monitoring ATX-a in real samples.

## 4. Conclusions

In summary, an aptamer-based colorimetric biosensor was developed for the detection of ATX-a in aqueous solutions using ATX-a-specific aptamers as the target recognition elements and AuNPs as colorimetric reporters. The change in AuNP aggregation was controlled by the interactions among the aptamer, AuNPs, and ATX-a in the presence of NaCl solution. The conformational change of the aptamer induced by the recognition of ATX-a caused a change in AuNP aggregation, resulting in a color change of the solution. This change was easily recorded using a UV/Vis spectrophotometer, and the obtained absorbance change generated a sensor signal for monitoring ATX-a. The relationship between the absorbance variation of the solution with and without ATX-a and the concentration of ATX-a was linear when the ATX-a concentration was in the range of 10 pM to 200 nM. Compared to previously reported methods (Table 1), the developed biosensor has a lower LOD (4.45 pM) and a wider linear range (10 pM to 200 nM). Moreover, the proposed aptasensor exhibited high selectivity toward ATX-a against other interferents and displayed satisfactory recovery in the detection of ATX-a in real samples. Therefore, the proposed colorimetric aptasensor is a promising alternative method that offers a simple, rapid, and sensitive tool for ATX-a detection in the environment.

## Figures and Tables

**Table 1:** Comparison with reported methods for anatoxin-a detection.

Methods	Linear Ranges	Limit of Detection	Reference
Electrochemistry	1–100 nM	0.5 nM	[1]
HPLC ^a^	0.3–9.078 μM	121 nM	[4]
GC–MS ^b^	2.5–200 ng/mL	2 ng/mL	[5]
Fluorescence	–	<3 nM	[6]
Electrochemiluminescence	1–1000 mg/L	0.34 mg/L	[7]
Colorimetric aptasensor	10 pM–200 nM	4.45 pM	Present study

^a^ HPLC, high-performance liquid chromatography. ^b^ GC–MS, gas chromatography–mass spectrometry.

**Table 2:** Determination of anatoxin-a (ATX-a) concentration in spiked lake water samples using our developed colorimetric aptasensor (*n* = 3).

Samples	Added ATX-a (nM)	Found ATX-a (nM)	Recovery (%)	RSD (%)
Lake water 1	0.1	0.08972	89.72	8.15
Lake water 2	0.5	0.4519	90.38	10.91
Lake water 3	1	1.0419	104.19	2.98
Lake water 4	50	56.215	112.43	6.37
Lake water 5	200	195.7	97.85	4.12

## Supplementary Materials

The following are available online at https://www.mdpi.com/article/10.3390/mi12121526/s1, Figure S1. (a) Optimization of the incubation time of aptamer with gold nanoparticles (AuNPs). (b) Optimization of the binding time of aptamer with anatoxin-a. Experimental conditions: concentration (C)_AuNPs_ = 4.7 nM, C_aptamer_ = 125 nM, C_NaCl_ = 40 mM, C_ATX-a_ = 100 nM.
