# Supplementary material for: A Simple and Ultrasensitive Colorimetric Biosensor for Anatoxin-a Based on Aptamer and Gold Nanoparticles
Source: Micromachines (Basel). 2021 Dec 8;12(12):1526. doi: 10.3390/mi12121526 (PMC8703760; doi:10.3390/mi12121526)
Supplement: Supplementary file 1 [file micromachines-12-01526-s001.zip › micromachines-1490647-supplementary.pdf]

Supplementary Material

A simple and ultrasensitive colorimetric biosensor for anatoxin-a based on aptamer and gold nanoparticles

Duy Khiem Nguyen[1] and Chang-Hyun Jang[1,*]

[1]Department of Chemistry, Gachon University, Seongnam-daero 1342, Sujeong-gu, Seongnam-si, Gyeonggi-do 13120, Republic of Korea.

[*]Corresponding author: Tel. +82-31-750-8555; fax: +82 31 750 8774
E-mail address: chjang4u@gachon.ac.kr (C.-H. Jang)

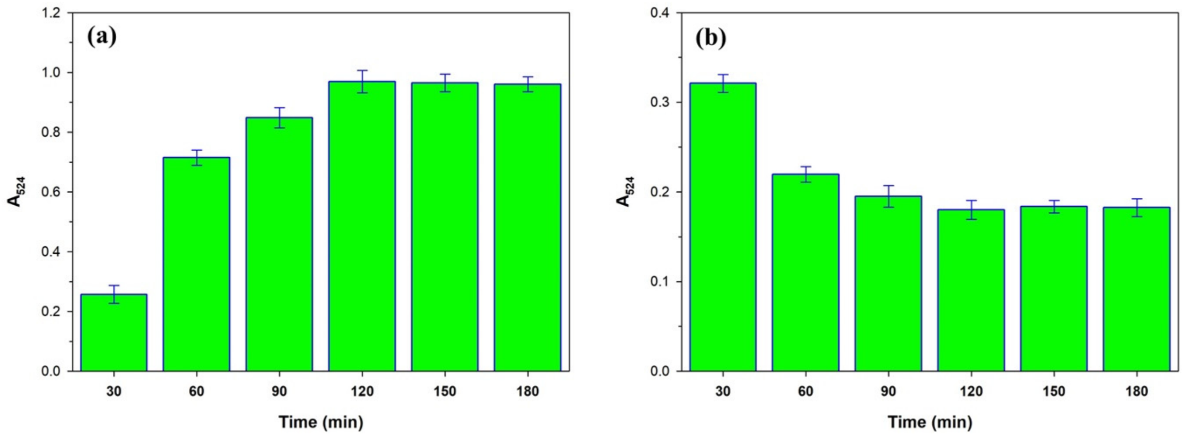

Figure S1. (a) Optimization of the incubation time of aptamer with gold nanoparticles (AuNPs). (b) Optimization of the binding time of aptamer with anatoxin-a. Experimental conditions: concentration $(C)_{AuNPs}$ = 4.7 nM, $C_{aptamer}$ = 125 nM, C_{NaCl} = 40 mM, $C_{ATX\text{-}a}$ = 100 nM.
